# A current perspective on antimicrobial resistance in Southeast Asia

**DOI:** 10.1093/jac/dkx260

**Published:** 2017-08-07

**Authors:** Raphaël M Zellweger, Juan Carrique-Mas, Direk Limmathurotsakul, Nicholas P. J Day, Guy E Thwaites, Stephen Baker, on behalf of the Southeast Asia Antimicrobial Resistance Network, Members of the Southeast Asia Antimicrobial Resistance Network, Elizabeth Ashley, Katinka de Balogh, Kevin Baird, Buddha Basnyat, Carolyne Benigno, Ladaporn Bodhidatta, Narisara Chantratita, Ben Cooper, David Dance, Mehul Dhorda, Rogier van Doorn, Gordon Dougan, Ngo Thi Hoa, Margaret Ip, Trevor Lawley, Cherry Lim, Thong Kwai Lin, Claire Ling, Yoel Lubell, Alison Mather, Florian Marks, Venkata Raghava Mohan, Paul Newton, Daniel Paris, Nicholas Thomson, Paul Turner, Oralak Serichantalergs, Frank Smithuis, Vanaporn Wuthiekanun, Nicholas White, Hsu Li Yang

**Affiliations:** 1 The Hospital for Tropical Diseases, Wellcome Trust Major Overseas Programme, Oxford University Clinical Research Unit, Ho Chi Minh City, Vietnam; 2 Centre for Tropical Medicine and Global Health, Nuffield Department of Clinical Medicine, Oxford University, UK; 3 Mahidol-Oxford Tropical Medicine Research Unit (MORU), Faculty of Tropical Medicine, Mahidol University, Bangkok, Thailand; 4 The London School of Hygiene and Tropical Medicine, London, UK

## Abstract

Southeast Asia, a vibrant region that has recently undergone unprecedented economic development, is regarded as a global hotspot for the emergence and spread of antimicrobial resistance (AMR). Understanding AMR in Southeast Asia is crucial for assessing how to control AMR on an international scale. Here we (i) describe the current AMR situation in Southeast Asia, (ii) explore the mechanisms that make Southeast Asia a focal region for the emergence of AMR, and (iii) propose ways in which Southeast Asia could contribute to a global solution.

## Introduction

In 1928 Alexander Fleming initiated a medical revolution by discovering penicillin, which was developed as a medication and cured previously life-threatening infections. In 1945, Fleming foretold the risks of antimicrobial resistance (AMR): ‘The time may come when penicillin can be bought by anyone in the shops. Then there is the danger that the ignorant man may easily under dose himself and by exposing his microbes to non-lethal quantities of the drug make them resistant.’^1^ Recently, the WHO stated that AMR in bacteria, viruses and parasites is ‘one of the greatest challenges in public health’,^2^ and could lead to ‘a post-antimicrobial era’ in which common infections may kill again.^3^ AMR, a natural evolutionary consequence of antimicrobial usage (AMU), is a global, multifactorial and complex problem intrinsically linked to human health and behaviour, but also entangled with animal health, food production, agriculture and the environment.^4^

Southeast Asia is a highly dynamic region characterized by rapid (yet uneven) economic development and has been proposed as an epicentre for emerging infectious diseases and AMR.^5^^,^^6^ This article aims to: (i) present the current AMR situation in Southeast Asia from the perspective of burden in humans, surveillance capacity, AMU and policy; (ii) explore the mechanisms that make Southeast Asia a focal region for the emergence of AMR; and (iii) propose ways in which Southeast Asia could contribute to a global solution.

AMR is a vast and complex problem, and rather than presenting a meta-analysis of AMR we aim to summarize and assess the key themes in regard to AMR in Southeast Asia. AMR in animals is inherently linked to AMR in humans, but will not be covered here as it has been recently been the subject of a review in Southeast Asia.^7^ Additionally, HIV, TB and malaria have been excluded as they present, to a large extent, different biological and public health issues. Therefore, this article focuses broadly on human infections in the low- and middle-income countries (LMICs) in Southeast Asia: Myanmar, Thailand, Cambodia, Lao PDR, Vietnam, Malaysia, the Philippines and Indonesia. Despite their disparities, these countries share many common characteristics with respect to AMR and there are repeating themes in human health in these countries.

## The current AMR situation in Southeast Asia

### (i) The human AMR burden in Southeast Asia

The WHO recently combined available worldwide data on AMR in seven common bacterial pathogens (*Escherichia coli*, *Klebsiella pneumoniae*, *Staphylococcus aureus*, *Streptococcus pneumoniae*, non-typhoidal *Salmonella*, *Shigella* spp. and *Neisseria gonorrhoeae*).^3^ The WHO report highlighted a lack of systematic data collection concerning AMR in Southeast Asia, and described the AMR problem as being ‘burgeoning and often neglected’.^3^ While the general consensus is that the AMR burden is large in Southeast Asia, a lack of standardized and comprehensive data prevents a precise quantification of AMR-associated morbidity, mortality and economic cost. There are, however, some country-specific examples; a recent study in Thailand showed that a total of 38481 patients with AMR hospital-acquired infections in 2010 died.^8^ This same study estimated that the antimicrobials necessary to treat AMR infections cost 202 million USD in 2010, and the total cost associated with AMR-related morbidity and premature deaths was 1.3 billion USD. A further study from Thailand suggested that 43% of deaths caused by hospital-acquired MDR bacterial infections in 2010 in Thailand were excess mortality due to MDR.^9^

### (ii) The current AMR surveillance capacity in Southeast Asia

Numerous networks contribute to AMR surveillance efforts in Southeast Asia (Table 1). However, the types and availability of data generated by these networks are highly heterogeneous and vary by country. Data regarding AMR are challenging to find for Cambodia, due to the scarcity of microbiological laboratories, but better data exist for Vietnam and Thailand.^9^^,^^10^ In general, AMR surveillance across the region is currently performed in healthcare settings, but hospital-associated AMR represents only a fraction of the total AMR burden, potentially leaving large knowledge gaps on the AMR burden in the community and in animals bred for food production.

**Table 1.** Networks contributing to surveillance efforts for AMR in Southeast Asia^a^

Name (year)	Description	Goal	Members
ANSORP: Asian Network for Surveillance of Resistant Pathogens (1996)	independent, non-governmental, not-for-profit international network for collaborative research on antimicrobial agents and infectious disease in Asia	to develop international strategies and action plans for effective control and prevention of AMR in Asia	113 hospitals in 65 cities in 14 countries: Saudi Arabia, Sri Lanka, India, China, South Korea, Japan, Hong Kong, Taiwan, Thailand, Vietnam, the Philippines, Malaysia, Singapore and Indonesia
VINARES: Vietnam Resistance	capacity-building initiative started after a report identified sub-optimal infection control, inadequate laboratory diagnostic capacity and inappropriate AMU as main drivers of AMR in Vietnam	to strengthen antimicrobial stewardship in Vietnam, particularly in the areas of (i) infection control and healthcare-associated infections, (ii) antimicrobial consumption and (iii) microbiological analysis and reporting capacity	16 hospitals throughout Vietnam. Collaboration between Vietnamese healthcare professionals, the Wellcome Trust Major Overseas Programme (WT-MOP) and Linköping University (Sweden)
Asia WT-MOPs: Wellcome Trust Major Overseas Programmes	local capacity building initiative focused on research, training and enhancing laboratory infrastructure	to improve patient clinical outcomes; to investigate infectious diseases transmission and susceptibility; to develop new tools to prevent, control and treat drug-resistant organisms; to enhance local public health policy	central units in Bangkok, Thailand (MORU) and in Ho Chi Minh City, Vietnam (OUCRU) (satellites in Lao PDR, Cambodia, Nepal, and Indonesia)
GARP: Global Antimicrobial Resistance Partnership (2008)	initiative launched to amplify the voice of LMICs at the AMR discussion table, funded by the Bill and Melinda Gates Foundation (member states are expected to sustain their activities after initial GARP funding)	to catalyse discussions between local experts in order to analyse the AMU and AMR situation, identify knowledge gaps, formulate locally relevant policies related to AMU and AMR in LMICs, promote those policies	active programmes in 8 countries: Kenya, India, Vietnam, South Africa, Mozambique, Tanzania, Nepal and Uganda
PulseNet Asia Pacific (2002)	network of laboratories performing molecular subtyping of bacteria and active in laboratory capacity building, training, quality assurance, protocol evaluation, standardization, and communication enhancement	to enable timely exchange of DNA fingerprinting data on pathogens causing foodborne outbreaks in the Asia Pacific region in order to enhance surveillance and provide early warning of outbreaks	Australia, Bangladesh, China, Hong Kong, India, Japan, Korea, Malaysia, New Zealand, the Philippines, Taiwan, Thailand and Vietnam
NARST: National Antimicrobial Resistance Surveillance Thailand (1998)	collaborative network initiated through funding from the WHO	to strengthen the surveillance programme for AMR and standardize laboratory practices in Thailand	collects data from 33 hospitals throughout Thailand
AMRCP: Thailand AMR Containment and Prevention	network created by academics and AMR thought leaders	to contain and limit the emergence and spread of AMR in Thailand by (i) estimating the AMR burden in Thailand, (ii) developing laboratory capacity, (iii) improving understanding of AMR and (iv) promoting responsible AMU (amongst others)	Thailand

a Information compiled from cited references and the networks’ respective websites.^8^^,^^29^^,^^30^^,^^78–80^

### (iii) Availability of antimicrobial usage data in Southeast Asia

As AMU is an important risk factor for the development of AMR, the collection of data on AMU in humans, agriculture and aquaculture is vital for understanding the emergence of AMR. Unfortunately, identifying reliable estimates for AMU in humans and animals across the region is extremely challenging. However, between 2000 and 2010, the per capita antimicrobial consumption decreased in Indonesia and the Philippines, but increased in Thailand, Malaysia and in Vietnam (between 2005 and 2010); no data were available for Myanmar, Cambodia and Lao PDR.^11^

AMU in primary healthcare is an important driver of regional AMR,^12^ but data for LMICs are scarce. It has been suggested that the proportion of patients receiving antimicrobials in primary healthcare has increased worldwide in the past decades,^13^^,^^14^ but nationwide estimates are lacking for many countries in Southeast Asia.^15^^,^^16^ Recently, a study in Malaysia aimed to fill this knowledge gap, and found that one in five patient encounters with a primary healthcare centre resulted in an antimicrobial prescription.^14^ The rate of antimicrobial prescribing was much higher in private (30.8%) than public (6.8%) clinics.

It has been estimated that veterinary AMU for food production will increase globally by 67% by 2030, and that one-third of this increase will be caused by a shift towards intensive livestock production in middle-income countries. Some of the largest increases in AMU (>200%) between 2010 and 2030 are predicted to arise in Myanmar and Indonesia.^17^ Despite this prediction, data on AMU in animal populations is particularly scarce, especially in LMICs. A survey from the World Organization for Animal Health (OIE) in 2012 showed that only 27% of its members had official systems to collect quantitative data on AMU in livestock production.^18^ Similarly, in a recent attempt to quantify AMU in animals worldwide, estimates regarding AMU in animals could be obtained from 32 high-income countries only.^17^

### (iv) Antimicrobial stewardship and policy in Southeast Asia

In 2011, the members of the WHO South East Asia Region (Bangladesh, Bhutan, Democratic People’s Republic of Korea, India, Indonesia, Maldives, Myanmar, Nepal, Sri Lanka, Thailand, Timor-Leste) signed the Jaipur Declaration, in which they recognized the seriousness of the AMR problem and committed to act in order to safeguard the efficacy of antimicrobial drugs. A year after signing this antimicrobial stewardship (AMS) initiative, 42% of the countries surveyed in Asia had national AMS standards (the worldwide proportion is 52%),^19^ and 63% of the hospitals surveyed in Asia reported having AMS standards (the worldwide proportion is 62%). As of April 2017, four countries in Southeast Asia (Cambodia, the Philippines, Thailand and Vietnam) have deposited national action plans against AMR in the WHO library of national action plans (http://www.who.int/antimicrobial-resistance/national-action-plans/library/en/). Notably, there is no universally accepted definition of AMS, but the term refers to the strategies, policies, guidelines and tools used to promote and increase the appropriate use of antimicrobials.^19^ The goal of AMS is to ensure effective treatment for patients with bacterial infection and to reduce unnecessary antimicrobial use in order to limit the development of AMR and preserve the efficacy of antimicrobials.

In Thailand, the Antimicrobial Smart Use (ASU) initiative was introduced in 2007 to promote the rational use of antimicrobials; this included a direct ban on the use of antimicrobials as growth promoters in animal food.^20^ Guided by the assumption that optimizing AMU requires a behavioural change, the ASU initiative attempted to rectify common misconceptions that patients have about antimicrobials, by clarifying that: (i) antimicrobials are not anti-inflammatory drugs; (ii) antimicrobials are potentially dangerous drugs; and (iii) upper respiratory tract infections, diarrheal diseases and simple cuts can be cured without antimicrobials. In addition, the ASU initiative tried to change prescription practices among health professionals, through education and training. In a further study in Thailand on how to improve AMS programmes in hospitals, the addition of trained infectious diseases clinical pharmacists to the normal consultation decreased inappropriate antimicrobial prescribing.^21^

## The drivers of AMR in Southeast Asia

**Figure 1.**
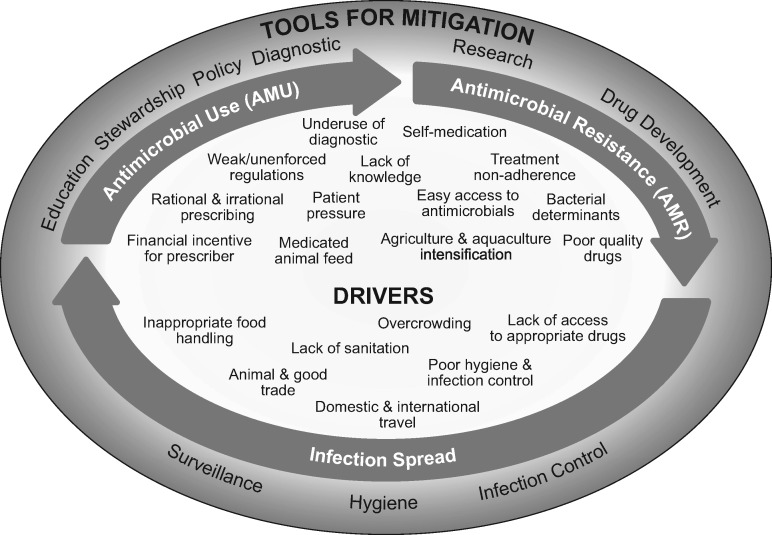
Schematic of the development, spread, drivers and tools for the mitigation of AMR. Drivers and tools for mitigation may influence any or all of AMU, AMR and infection spread. Their location on the schematic does not imply anything about where they play a role.

The drivers that are likely to have an impact on AMR emergence and transmission in Southeast Asia are outlined below and displayed in Figure 1; we have summarized these into five main areas.

### (i) Economic development and population growth stimulate antimicrobial demand

Southeast Asia has recently experienced tremendous socioeconomic development and population growth (Table 2).^6^ The rapid development of transport infrastructures has improved the mobility of people, animals and goods.^5^ Prosperity and population growth have increased demand for animal protein. The per capita consumption of animal protein increased by 45% between 1990 and 2000 in the Mekong region (Cambodia, Lao PDR, Thailand and Vietnam) alone,^6^ and exports have increased correspondingly. Meat and fish production systems have become more intensive to meet this demand.^22^ Between 1995 and 2013/14, livestock production doubled across the region, and fish production tripled (Table 2). Vietnam is currently the third largest producer of aquaculture products globally, behind China and India.^23^^,^^24^ Presently, large-scale, intensive livestock and fish production systems operate alongside traditional fishing and farming practices, as well as mixed aquaculture–livestock systems, all of which rely heavily on antimicrobial agents.^6^^,^^17^^,^^24–27^ As estimates suggest that AMU for food production surpasses AMU in humans,^17^ this increase in demand for animal protein is likely to contribute to AMR via an increase in AMU. A transition to more intensive farming methods has increased the demand for antimicrobials, and market reforms have improved their availability. As a rule AMU in animals is poorly regulated (or rules are not enforced), which therefore amplifies the risk of excessive or inappropriate AMU.^6^^,^^23^^,^^28–32^

**Table 2.** Socioeconomic development in Southeast Asia 1995–2014

	Cambodia			Indonesia			Lao PDR			Malaysia		
Indicator^a^	1995	2014^b^	% change	1995	2014^b^	% change	1995	2014^b^	% change	1995	2014^b^	change

Population (millions)	10.7	15.3	43	197.0	254.5	29	4.9	6.7	38	20.7	29.9	44
Life expectancy (years)	55	68	24	65	69	6	56	66	18	72	75	4
Infant mortality rate (per 1000 live births)	88	26	–70	51	24	–53	97	52	–46	12	6	–50
GDP per capita (USD)	322	1095	240	1026	3492	240	363	1794	394	4280	11307	164
Livestock production index 1995–2013^c^	69.8	89.6	28	79.5	139.9	76	72.9	125.8	73	88.2	138.8	57
Total fisheries production (millions of tons)	0.11	0.75	562	4.39	20.88	376	0.04	0.15	274	1.25	1.99	59
CO_2_ emission 1995–2011 (tons per capita)	0.1	0.3	200	1.1	2.3	109	0.1	0.2	100	5.8	7.9	36

	Myanmar			Philippines			Thailand			Vietnam		
	
Indicator^a^	1995	2014^b^	% change	1995	2014^b^	% change	1995	2014^b^	% change	1995	2014^b^	change

Population (millions)	44.7	53.4	20	69.8	99.1	42	59.3	67.7	14	72.0	90.7	26
Life expectancy (years)	60	66	10	66	68	35	70	74	6	72	76	6
Infant mortality rate (per 1000 live births)	69	41	–41	34	23	–32	24	11	–54	31	18	–42
GDP per capita (USD)	ND	1204	ND	1061	2873	171	2856	5977	109	288	2052	613
Livestock production index 1995–2013^c^	33.4	191.9	475	64.2	126.5	97	99.2	130.2	31	49.5	147.4	198
Total fisheries production (millions of tons)	0.82	5.05	513	2.81	4.69	67	3.59	2.70	–25	1.47	6.33	329
CO_2_ emission 1995–2011 (tons per capita)	0.2	0.2	0	0.9	0.9	0	2.8	4.5	61	0.4	2.0	400

ND, not determined.

a Source: World Bank, http://www.worldbank.org.

b Except for Livestock production index, which is given for the period 1995–2013.

c Livestock production index includes meat and milk from all sources, dairy products such as cheese, and eggs, honey, raw silk, wool, and hides and skins; reference year 2004–2006 = 100.

### (ii) Antimicrobial usage in human medicine

Antimicrobials are amongst the most frequently prescribed drugs in human medicine worldwide.^33^ In Vietnam, antimicrobials account for >50% of drugs used in human medicine,^28^ and are the most commonly sold drugs in pharmacies.^32^ Early symptoms of infections with many bacteria, viruses and parasites are comparable, making it difficult to determine the aetiology of non-specific conditions such as fever, diarrhoea or respiratory tract infections. Rapid diagnostic tests that can discriminate between viral and bacterial infections are rarely used in LMICs.^34^ As bacterial infections can progress quickly, antimicrobials are often prescribed as a safeguard without confirmation of the aetiology, and, therefore, are often unnecessarily used for non-bacterial infections. A study from Vietnam found that one-third of hospitalized patients received an antimicrobial in the absence of a correct medical indication;^35^ this is partly because many hospitals in Vietnam lack adequate capacity and sufficiently trained staff to isolate the infecting bacteria and determine their antimicrobial susceptibility profiles.^28^^,^^29^

A common theme across Southeast Asia is self-medication (without prescription): antimicrobials are available ‘over the counter’ in all of the selected countries, and self-treatment is generally cheaper and more convenient than visiting a healthcare facility.^28^^,^^36^^,^^37^ Studies in Vietnam found that 90% of antimicrobials were sold without prescription in pharmacies despite a prescription-only regulation in the Drug Law existing since 2005, and in Indonesia >90% of pharmacies fulfilled antimicrobial requests without a prescription.^30^^,^^32^^,^^38^^,^^39^ Pharmacists are under pressure to satisfy the demand for antimicrobials for fear of losing customers, as antimicrobials represent a large fraction of their income, and prescription-only regulations are rarely enforced.^32^ A comparable study of pharmacies in Thailand demonstrated that shops often sold antimicrobials to customers complaining of a cold, acute diarrhoea or dysuria.^40^

### (iii) Antimicrobial awareness, knowledge and prescribing practices

The risk of inappropriate AMU through self-medication, unregulated access and irrational prescription is magnified by a sub-optimal understanding of antimicrobials and their use amongst the public, drug sellers and doctors in Southeast Asia.^32^^,^^38^^,^^41^^,^^42^

A lack of knowledge among the public stimulates irrational demand for antimicrobials and/or treatment non-adherence, which are two key drivers of AMR. A study from Malaysia found that over half of the patients discontinued their antimicrobial treatment once symptoms disappeared.^43^ Treatment non-compliance was also associated with limited knowledge about antimicrobial function and low AMR awareness but sometimes arose due to limited financial means.^43^ As a Vietnamese pharmacist explained: ‘Patients want antimicrobials, but can only afford half a cure, and as a consequence under-dose themselves.’^32^

In a recent survey of doctors from the Lao PDR,^44^ 96.6% considered AMR to be a serious problem, but 29.8% believed that prescribing unnecessary antimicrobials was harmless. Furthermore, 76% of doctors agreed that antimicrobials were overused in hospitals and the community, but only 47.1% thought that it was a problem in their own hospital. More than half of the doctors had no information regarding local antimicrobial susceptibility patterns for the aetiological agents of typhoid fever and hospital-acquired pneumonia. These data were echoed in Malaysia, where 83% of medical students surveyed acknowledged that AMR was a national problem, but only 63% thought it happened in their hospital.^45^ Again, comparable results were obtained in Cambodia.^46^

The aforementioned study in Lao PDR also confirmed the difficulty of prescribing an appropriate antimicrobial (recognized by 72.5% of the doctors).^44^ Almost all (96.6%) doctors surveyed would welcome more training on antimicrobial prescribing, as would 88% of medical students surveyed in Malaysia.^45^ In a further study from Malaysia, 21.6% of doctors acknowledged that even when they thought antimicrobials were unnecessary, they may prescribe them to comply with patients’ requests.^47^ Half of the doctors surveyed in Lao PDR agreed that patients’ expectations influenced antimicrobial prescribing.^44^ In Thailand, unnecessary antimicrobial prescribing was associated with the prescriber’s low understanding of antimicrobials and their usage, as was a perceived pressure from patients expecting antimicrobial treatment.^20^

A further study conducted in Malaysia indicated that despite the existence of national antimicrobial guidelines, antimicrobial overprescribing is common in primary healthcare facilities, particularly in private clinics.^14^ Upper respiratory tract infections (URTIs) accounted for almost half (49.2%) of the prescriptions, illustrating unnecessary AMU for a condition that is often of viral aetiology. Antimicrobials were prescribed to 46.2% of the patients diagnosed with URTI in Malaysia but, in comparison, only to 17% and 5% of comparable patients in the Netherlands and Hong Kong, respectively.^14^ In LMICs (including two Southeast Asian countries), AMR has been found to be significantly associated with out-of-pocket health expenses in the public sector.^48^ The authors of that article speculated that higher costs in the public sector divert patients towards the private sector, where antimicrobials are prescribed more frequently due to stronger financial incentives (supplier-induced demand). Indeed, in Vietnam many patients self-medicate or use the private sector as it is cheaper than the public sector.^36^ This problem may persist, because out-of-pocket health spending in the region is projected to rise to 43.5% by 2040, more than twice the predicted world average of 20.6%.^49^

### (iv) Antimicrobials in agriculture and aquaculture

Antimicrobial overuse, misuse and a lack of awareness are also common in animal production across the region; for a summary of available data on AMU in animal production in Southeast Asia readers are directed to a recent review.^7^ Briefly, livestock and fish producers in Southeast Asia are under pressure to satisfy both a growing domestic demand and the export industry, and rely heavily on antimicrobials. Antimicrobial agents are routinely used in livestock production in Southeast Asia to treat infections (therapeutic use), to prevent infections (prophylactic use), to treat asymptomatic animals belonging to a group where other animals have disease symptoms (metaphylactic use), and at sub-therapeutic concentrations to promote growth, a practice that is increasingly controversial (growth promoters).

In Vietnam, antimicrobials for animal production are available over the counter, and veterinary pharmacists are a major source of advice for farmers.^25^^,^^26^ Farms and aquaculture systems producing for the domestic market benefit from much looser regulations compared with those producing for export, particularly to Europe and the United States.^23^^,^^28^

Studies in Vietnam have found that antimicrobials are predominantly used to prevent rather than to treat infections in poultry and pig production systems;^25^^,^^26^ additionally, it has been reported that AMU in chicken production in the Mekong delta is approximately six times greater than in many European countries.^25^ In addition, commercial pigs and poultry feeds are routinely medicated.^27^ In contrast with human medicine, where subjects are treated individually, antimicrobials are normally administered to groups of animals (herds/flocks/ponds) in animal production.^31^^,^^50^ Importantly, according to the Food and Agriculture Organization (FAO), all classes of antimicrobials important for human medicine are used in animals in Southeast Asia.^26^ In Vietnam, antimicrobials considered of critical importance for human medicine by the WHO are used in poultry and pig farms (penicillins, third-generation cephalosporins, quinolones, aminoglycosides, polymyxins, and macrolides).^25^^,^^51^^,^^52^^,^^53^

In Southeast Asia, the use of antimicrobials is also normal in intensive aquaculture, as well as integrated agriculture–aquaculture systems where humans, vegetable/rice fields, livestock and aquaculture ponds are in close proximity.^6^^,^^54^ For example, studies have revealed that antimicrobials (including critically important antimicrobials for human use) are routinely used in >70% of aquaculture systems in Vietnam and Thailand.^23^^,^^51^^,^^55^^,^^56^ Antimicrobials in aquaculture are commonly administered in feed, and therefore given indiscriminately to healthy and infected shrimp or fish.^23^^,^^24^ A study found that 26.9% of fish and shrimp samples bought in local markets in Vietnam contained antimicrobial residues.^23^ In addition, many antimicrobial products used in Vietnamese aquaculture did not contain the concentrations of antimicrobials presented on the label, or provided erroneous dilution information.^57^ Farmers are usually ill-informed about antimicrobials, and totally unaware of the prescribing regulations.^23^

AMR organisms and genetic elements encoding for resistance generated and maintained on farms may subsequently transmit to humans via direct contact with animals, consumption of foods of animal origin and/or their dissemination through animal waste.^58^^,^^59^ The problem of AMR gene transfer is likely exacerbated by poor sanitation and the lack of appropriate waste treatment and biocontainment in many farms in Southeast Asia.^54^^,^^55^^,^^60^ Human and livestock excreta are often used to fertilize fish ponds, creating the optimal context for transfer of AMR genes or AMR bacteria between animal species, and subsequent contamination of water sources. As an illustration, 87% of the *E. coli* isolated from the Matang mangrove estuaries in Malaysia were resistant to at least 1 of 15 antimicrobials tested, and 34% were resistant to three or more antimicrobial classes.^61^ The authors postulated an association between the high level of multidrug resistance and the proximity to fishing villages lacking adequate sewerage and sanitation.

### (v) Drug access and quality

Access to antimicrobials has improved regionally; this has both positive (treating illnesses) and negative (inappropriate AMU, a major driver of AMR) consequences. It is worth noting that lack of timely access to good-quality antimicrobials remains a reality for many.^62^ In a study from Lao PDR, one in five doctors declared that their antimicrobial prescribing was more influenced by what was available at the hospital than by the presumed aetiological agent,^44^ and 38.3% of the participants considered that some of the antimicrobials available at their hospital were of poor quality.

It is generally difficult to ascertain the overall quality of antimicrobials available in Southeast Asia. According to the WHO, up to 10% of all drugs worldwide are counterfeit. Counterfeit drugs include drugs that are incorrectly dosed, contain the wrong active ingredient, contain no active ingredient, are of sub-standard quality or are wrongly packaged. Antimalarials and antibacterials are thought to be the most frequently counterfeited drugs.^63^ Worldwide sales of counterfeit drugs generate an estimated $75 billion a year,^64^ and Southeast Asia produces more than three-quarters (78%) and consumed almost half (44%) of counterfeit antimicrobials globally.^65^ A recent review confirmed that almost half of all counterfeit antimicrobials were found in Asia.^63^ A further study found that 31% of drugs tested had an active pharmaceutical ingredient at least 15% lower (or higher) than indicated on the label.^66^ A further study from Cambodia showed that 14.5% of drugs tested (including antimicrobials) were unacceptable with respect to the quantity of active ingredient.^67^ Below-par drug quality is particularly alarming in the context of AMR, as antimicrobial drugs in which the active pharmaceutical ingredient is of low quality or dosage expose pathogens to sub-therapeutic doses, which then favours the development of resistance.

## Solutions to the crisis: surveillance, diagnostics, stewardship and qualitative research

While it is difficult to quantify precisely the relative contribution of each driver to the development and spread of AMR throughout Southeast Asia, acting locally on defined drivers and finding region-specific solutions to mitigate AMR should be a priority. Members of the public, of the scientific and medical communities, local and national authorities as well as the international community can and should contribute to the solution. The United Nations has recognized the need to act and, in a resolution adopted in October 2016, reaffirmed that ‘the blueprint for tackling AMR is the World Health Organization Global Action Plan on AMR’.^68^ In addition, the resolution confirms the commitment to develop national plans on AMR, and mobilize the necessary resources to do so. In response the WHO has issued the ‘five pillars’ of the WHO Global Action Plan on AMR.^69^ The aims of the WHO Global Action Plan on AMR are: (i) to improve awareness and understanding of AMR through communication, education and training; (ii) to strengthen the knowledge and evidence base through surveillance and research; (iii) to reduce the incidence of infection through effective sanitation, hygiene and infection prevention measures; (iv) to optimize the use of antimicrobial medicines in human and animal health; and (v) to develop the economic case for sustainable investment that takes account of the needs of all countries, and increase investment in new medicines, diagnostic tools, vaccines and other interventions.^69^ Outlined below are some Southeast Asia-focused comments on the pillars of the WHO Global Action Plan on AMR.

### (i) The need for collaborative surveillance networks

Considerable surveillance infrastructure, capacity and know-how already exist in Southeast Asia, but are highly variable across countries and sectors. In general, surveillance capacity occurs in many hospitals, but the animal production sector lags behind and knowledge gaps in the community remain. Collaboration and coordination between existing surveillance networks could develop synergies, avoid redundancy and promote standardized collection and analysis of samples. Sample collection and analysis (particularly determination of AMR profile) and the dissemination of results should follow the recommendations of WHO’s Global Antimicrobial Resistance Surveillance System (GLASS),^70^ in order to ensure comparability with other surveillance networks worldwide. Standardized reporting of results via a common platform would permit real-time information sharing, in a form that is clear and useful for the public, health professionals and policymakers.

Sample collection, analysis and result dissemination should remain compatible with the implementation of new technologies (gene arrays, PCR amplification, WGS, etc.) for future research projects, such as elucidating gene flow between species and understanding the sinks and sources of AMR genes and organisms. PCR amplification of AMR genes and WGS are emerging into mainstream diagnostic laboratories in high- and middle-income countries. Whilst currently expensive and technically demanding, WGS of sentinel organisms within a Southeast Asia surveillance programme will become a key tool for understanding how AMR emerges and spreads across the region and beyond. In order to create a comprehensive picture of AMR, longitudinal sample collection for surveillance should extend to healthcare settings, the community, animal production and agriculture.

### (ii) The need for rapid diagnostics to improve antimicrobial prescribing

The availability of a rapid diagnostic test that can differentiate between viral and bacterial infection in primary point-of-care facilities in Southeast Asia would not only improve clinical management of undifferentiated fever, but also help to optimize antimicrobial prescription, thereby limiting irrational AMU. Recent studies in Lao PDR, Cambodia, Thailand and Vietnam have demonstrated that rapid C-reactive protein (CRP) tests were able to differentiate between viral and bacterial aetiology in patients with non-specific fever, and were a comparatively inexpensive way to reduce inappropriate AMU in basic healthcare facilities without a laboratory.^34^^,^^71^^,^^72^ An additional investigation showed that the concurrent use of two point-of-care rapid tests (urine dipstick and microscopy) improved antimicrobial prescribing in adults with urinary tract infections at the Thailand–Myanmar border.^73^

Diagnostic capacity (microbiological culture and AMR profiling) and/or rapid testing (to differentiate viral versus bacterial aetiology) should be improved in Southeast Asia. Capacity building, staff training and education would increase the prevalence of testing, thereby causing a shift from (mostly) empirical treatment towards evidence-based prescribing. In addition, there is a critical need to advertise broadly the importance of testing as a tool for rational diagnostics, and to understand the local, national and regional barriers to better diagnostic testing.

### (iii) The need for better awareness, education and stewardship

Studies suggest that education targeted at providers and consumers can contribute to reducing antimicrobial overuse.^74^^,^^75^ A recent survey assessing data from 55 countries (including Indonesia, Lao PDR and Cambodia) demonstrated that the presence within the health ministry of a department promoting rational use of medicines, a national strategy to contain AMR, a national drug information centre, and drug committees in hospitals and provinces were all associated with lower AMU.^76^ Other policies that have been proposed to optimize antimicrobial prescribing include (enforcement of) a blanket ban on over-the-counter antimicrobials, and preventing prescribers from selling antimicrobials for profit (to prevent supplier-induced demand).

AMS is a key component of the fight against AMR. More AMS initiatives should be developed in Southeast Asia, but additional research is necessary to understand which elements of a stewardship programme are the most effective, as this remains a matter of considerable debate.^77^ Coordination and standardization of the AMS initiatives throughout the region could develop synergies and help compare the effect of stewardship initiatives. Better communication between stewardship initiatives would allow sharing of the lessons learned by more mature AMR programmes.

### (iv) The need for social research

AMU and AMR have a large behavioural component. Antimicrobials are seen as ‘wonder drugs’, but their true positive and negative potentials are misunderstood. Social research would generate invaluable insight for understanding behaviours underlying irrational prescribing by healthcare professionals, irrational demand by the public and irrational use by farmers. Therefore, social research is an essential priority for achieving the changes necessary to optimize AMU. Similarly, understanding why antimicrobial prescribing is often performed without diagnostic testing will begin to restrict the inclination to use antimicrobials indiscriminately. There is an urgent need to assess what combinations of cost, reagent accessibility, time and training are restricting better diagnostic approaches. Lastly, understanding the reasons for self-medication, treatment non-adherence and the reasons why antimicrobials are sold without prescription is crucial.

Beyond morbidity, mortality and economic cost, qualitative research will be particularly important to estimate the wider societal cost of AMR, including the loss of efficacy of antimicrobials (and the effect of this on modern medicine), as well as deleterious effects of AMR on human capital, labour force, gross domestic product and economic growth.

Finally, research will prove key to understanding and estimating the relative contribution of the different drivers of the development of AMR. Additional research will be necessary to design effective interventions and estimate their ability to curb AMR. This novel information will be vital to galvanize political will, convince regulators to modify policy, and persuade people to adapt their behaviour.

## Conclusions

AMU and AMR are increasing in Southeast Asia, driven by rapid intensification of food-production systems, loosely regulated access to antimicrobials, poor awareness with respect to antimicrobials (from the public, health professionals and farmers), widespread irrational prescribing and self-medication, and an abundance of low-quality or counterfeit drugs. Combined with a high prevalence of infectious disease and weak diagnostic capacity, particularly in primary healthcare settings, Southeast Asia is a global hub for AMR, and contributes to the global spread of AMR as bacteria are readily transported to other parts of the world by international travellers, and by international trade of animals and goods. Across the region, there is generally good technical capacity to conduct laboratory testing and bacterial diagnostics. However, further work is needed to reach a consensus as to how to move forward with surveillance systems both for AMU and AMR in humans and in animal production systems.

While concerted global action across multiple sectors is required to tackle the spread of AMR, we suggest that Southeast Asia should be a crucial location for generating a global solution by: (i) improving, coordinating and developing surveillance capacity to detect outbreaks and monitor trends; (ii) promoting the widespread use of diagnostic tests to support evidence-based prescribing; (iii) developing stewardship initiatives to raise awareness, educate and optimize AMU; and (iv) performing social research to understand the human drivers of irrational prescribing and irrational demand for antimicrobials. All these activities would contribute to optimize and reduce AMU, curb AMR spread and ultimately improve population health while preserving the efficacy of existing antimicrobial drugs.
